# Bioactive adrenomedullin dynamics and microcirculatory changes in a porcine model of hemorrhagic shock

**DOI:** 10.1186/s40635-026-00976-4

**Published:** 2026-10-13

**Authors:** Carolin Vekla, Oliver Hartmann, Sebastian O. Decker, Maik von der Forst, Florian Uhle, Alexander Studier-Fischer, Berkin Özdemir, Felix Nickel, Stephan Katzenschlager, Frank Weilbacher, Thorsten Brenner, Erik Popp, Markus A. Weigand, Karsten Schmidt, Maximilian Dietrich, Daniel Gruneberg

**Affiliations:** 1 Department of Anesthesiology, Heidelberg University Hospital, 69120 Heidelberg, Germany; 2 SphingoTec GmbH, Neuendorfstr. 15A, 16761 Hennigsdorf/Berlin, Germany; 3 Department of Urology, Mannheim University Hospital, 68135 Mannheim, Germany; 4 Department of General, Visceral and Transplantation Surgery, Heidelberg University Hospital, 69120 Heidelberg, Germany; 5 Department of General, Visceral and Transplantation Surgery, University Hospital Hamburg-Eppendorf, 20246 Hamburg, Germany; 6 Department of Anesthesiology and Intensive Care Medicine, University Hospital Essen, University Duisburg-Essen, 45147 Essen, Germany

**Keywords:** Hemorrhagic shock, Resuscitation, Microcirculation, Adrenomedullin, Endothelial damage

## Abstract

**Background:**

Hemorrhagic shock is a leading cause of preventable trauma death, marked by impaired oxygen delivery and endothelial dysfunction. Bioactive adrenomedullin (bio-ADM) regulates endothelial barrier integrity, but its dynamics during hemorrhagic shock and resuscitation remain unclear. This secondary analysis of a porcine hemorrhagic shock model investigated bio-ADM and its association with macro- and microcirculatory parameters during resuscitation. All animals received crystalloid infusion (10 mL/kg/h) from anesthesia induction onward. Shock was induced by blood withdrawal to maintain MAP at 40 ± 5 mmHg for 60 min (T5), before randomization to control (n = 7), fluid (n = 6), or noradrenaline (n = 7) groups, with intervention groups titrated to MAP targets of 65 then 90 mmHg (60 min each). Macrocirculation was assessed via vital signs and pulse contour analysis; microcirculation via hyperspectral imaging (tissue oxygenation, hemoglobin, water index).

**Results:**

Fluid resuscitation improved macrohemodynamics and microcirculatory oxygenation versus control and noradrenaline, which failed to sustain perfusion and impaired microcirculatory oxygenation. Bio-ADM increased after shock induction (p < 0.001), peaking at T9 (120 min of therapy). At T5, levels were comparable among control (16.8 [13.6–18.9] pg/ml), fluid (15.65 [15.43–16.55] pg/ml), and noradrenaline (14.3 [7.85–18.95] pg/ml). Thereafter, bio-ADM continued to rise in control (19.7 [17.4–32.6] pg/ml) and noradrenaline (21.2 [14.4–33.45] pg/ml), while stabilizing in the fluid group (12.05 [10.65–15.18] pg/ml); accordingly, the T5–T9 increase was smaller in the fluid group than in control (p = 0.022) or noradrenaline (p = 0.003). The fluid group received ~3-fold more fluid by T9. Higher bio-ADM correlated with lower MAP, higher lactate, and lower ScvO₂, but not oxygen delivery (DO₂); a higher tissue water index correlated with a smaller rise.

**Conclusions:**

Bio-ADM increased during hemorrhagic shock, extending previous observations from cardiogenic and septic shock, and represents a comparatively non-specific marker of endothelial and microcirculatory stress rather than specific to any single shock etiology. Effective resuscitation was associated with stabilization, rather than a clear decrease, of bio-ADM levels, though this coincided with higher fluid administration, and dilution cannot be excluded. Whether bio-ADM offers therapeutic utility to guide resuscitation remains unclear, warranting further prospective evaluation in animal and human studies.

**Supplementary Information:**

The online version contains supplementary material available at https://doi.org/10.1186/s40635-026-00976-4.

## Background

Hemorrhagic shock remains one of the leading causes of preventable death following trauma, characterized by a critical reduction in tissue oxygenation due to decreased cardiac output through hypovolemia [1]. The reduced blood flow leads to inadequate oxygen supply, triggering cellular injury through impaired oxidative metabolism and consequent ATP depletion, which is essential for cellular pump functions and membrane integrity. As a result, prolonged hypoperfusion culminates in cellular death and organ dysfunction.

The pathophysiology of hemorrhagic shock involves two key injury phases: ischemic injury from low blood flow and reperfusion injury upon restoration of circulation. The latter phase is marked by the release of oxygen radicals that disrupt cellular membranes, inciting cytokine release and neutrophil activation. This inflammatory cascade exacerbates tissue damage, notably impacting the endothelium, whose impairment manifests as “endotheliopathy of trauma” [2]. This syndrome is driven by the shedding of the endothelial glycocalyx and is associated with coagulopathy, inflammation, compromised blood-organ barriers, and dysregulated vasoreactivity. Collectively, these changes contribute to increased microvascular permeability, edema, and thrombus formation, all of which further impair tissue perfusion and elevate the risk of organ failure​ [3]. Emerging research has highlighted the endothelium’s pivotal role in trauma pathophysiology, with a growing focus on targeting endothelial stability as a therapeutic strategy [4–6].

Bioactive Adrenomedullin (bio-ADM) is a small peptide hormone produced by endothelial and smooth muscle cells. It plays a critical role in maintaining endothelial barrier integrity by reducing actomyosin contraction, which decreases stress on tight junctions and enhances the stability of adherens junctions, leading to reduced vascular permeability [7]. Bio-ADM also induces vasodilation, which increases cardiac output and regulates fluid-electrolyte balance, highlighting its multifaceted role in vascular homeostasis [8]. Increased plasma bio-ADM levels have been observed in congestive heart failure, acute heart failure, sepsis and cardiogenic shock [9, 10]. In sepsis, the increase in bio-ADM likely is a failing endogenous protective response to endothelial damage [7]. However, high bio-ADM levels are also associated with exacerbated vasodilation and correlate with greater disease severity and mortality, potentially indicating a breakdown of compensatory mechanisms [11, 12].

Despite bio-ADM’s established role in other forms of shock, its specific function during hemorrhagic shock remains underexplored. This secondary analysis examines data from a previously conducted study of hemorrhagic shock in a porcine model, focusing on bio-ADM’s behavior across different shock stages and therapeutic interventions [13]. Additionally, this analysis investigates the relationship between bio-ADM levels and various markers of macro- and microcirculation, emphasizing the potential of bio-ADM as a therapeutic target for monitoring and improving endothelial function and microcirculation in response to hemorrhagic shock treatment.

## Material and methods

### Study design and ethical approval

This secondary analysis is based on data from a previously conducted study on hemorrhagic shock in a porcine model [13]. The study was approved by the appropriate governmental body (Regierungspräsidium Karlsruhe, file reference G-261/19) and conducted in accordance with the European law on the protection of animals used for scientific purposes (EU-Directive 2010/63).

The primary endpoint of this study was the temporal course of plasma bio-ADM levels during hemorrhagic shock and subsequent resuscitation, with a focus on differences between three therapeutic strategies (no therapy, vasopressor therapy, and fluid therapy). Secondary endpoints included the correlation of bio-ADM levels with hemodynamic parameters, surrogate markers of tissue perfusion, and hyperspectral imaging (HSI)-derived parameters of microcirculatory function.

### Animals and experimental protocol

Animal handling, anesthesia, and monitoring followed established protocols, as detailed previously in Dietrich et al. [13]. In brief, the original study involved 27 pigs (*Sus scrofa* subjected to a double-hit model combining major abdominal surgery and hemorrhagic shock. Throughout the experiment (from immediately after anesthesia induction until death, all pigs received standardized background crystalloid infusion at 10 mL/kg/h (Sterofundin Iso®; B. Braun®, Melsungen, Germany.

Following midline laparotomy, major abdominal surgery was simulated via splenectomy and stomach mobilization. Hemorrhagic shock was then induced by controlled blood withdrawal to maintain a mean arterial pressure (MAP) of 40±5mmHg for 60 min. The animals were then randomly divided into three groups: a control group (CG), which received no further intervention, and two intervention groups. For both intervention groups, MAP targets were set at 65 mmHg for the first 60 min, followed by 90 mmHg for the subsequent 60 min. The fluid group (FG) received continuous fluid infusion, whereas the noradrenaline group (NG) received noradrenaline. Both interventions were titrated at the investigators’ discretion to achieve the predefined MAP targets.

### Data collection

**Fig. 1:**
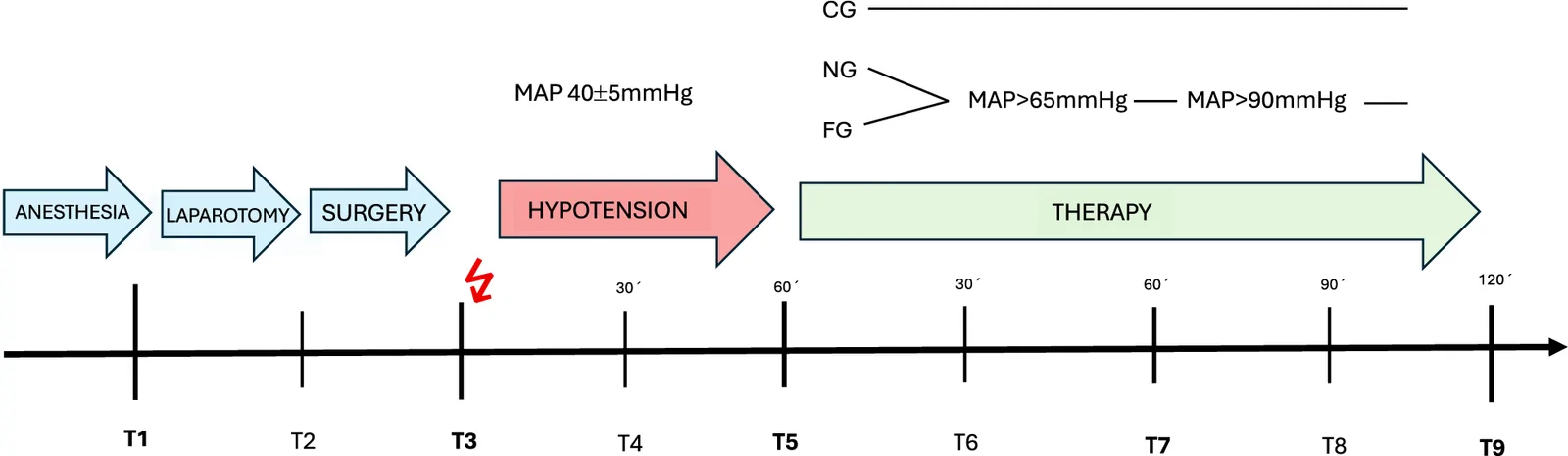
Timeline of hemorrhagic shock experiment and resuscitation interventions. The timeline illustrates the main experimental phases: Baseline-assessment after induction of anesthesia (T1), after laparotomy (T2), and immediately post-surgery (T3). Hemorrhagic shock with MAP 40±5mmHg was induced after T3 and maintained for 60min without treatment (T4–T5). Resuscitation therapy (T5–T9) comprised three groups: control (CG), noradrenaline (NG), and fluid group (FG). In NG and FG, MAP targets were >65mmHg during the first 60min and >90 mmHg during the subsequent 60min. Measurements were taken every 30min

Primary data collected in the initial study included monitoring of vital parameters as well as advanced hemodynamic monitoring using an arterial PiCCO catheter (PULSION Medical Systems SE, Feldkirchen, Germany) at nine predefined timepoints (Fig. 1).

Simultaneously, microcirculatory status at the ear and the kidney was recorded using hyperspectral imaging via the Tivita® Tissue System (Diaspective Vision GmbH, Am Salzhaff, Germany). Additionally, blood samples were taken to measure venous and arterial blood gases and bio-ADM.

### Hyperspectral imaging

Hyperspectral imaging was performed using the Tivita® Tissue System (Diaspective Vision GmbH, Germany). A more detailed technical description can be found elsewhere [14, 15]. The system captures 100 wavelengths to assess tissue parameters through several indices:

- **Tissue Oxygenation (StO₂):** Measures hemoglobin oxygen saturation in superficial capillaries (~ 1 mm depth) within 500–650 nm and 700–815 nm, expressed as a percentage (0–100%).
- **NIR Perfusion Index (NIR):** Reflects oxygen saturation in deeper tissue layers (4–6 mm) using wavelengths 655–735 nm and 825–925 nm, reported in arbitrary units (0–100).
- **Tissue Hemoglobin Index (THI):** Indicates distribution of oxygenated and deoxygenated hemoglobin up to 3 mm depth, measured at 530–590 nm and 785–825 nm (arbitrary units 0–100).
- **Tissue Water Index (TWI):** Assesses relative water content in tissue (1–3 mm depth) within 880–900 nm and 955–980 nm, also in arbitrary units (0–100).

Images were taken from the ear and kidney, selecting the largest uniform areas possible. Standardized distance and lighting conditions were maintained to ensure consistency, with the setup monitored for stray light.

### Bio-ADM assessment

For this secondary analysis, we specifically examined bio-ADM levels at various shock and resuscitation stages. Additionally, correlations between bio-ADM and markers of both macro- and microcirculatory function were analyzed to assess bio-ADM’s role in hemorrhagic shock and evaluate its relationship with different resuscitation strategies.

All bio-ADM measurements were performed blinded to clinical data at the laboratories of SphingoTec GmbH (Hennigsdorf, Germany), using a previously described one-step immunoassay [16]. Briefly, specific monoclonal antibodies directed against the middle portion of bio-ADM and the amidated C-terminus enable direct quantification of active bio-ADM. For a valid measurement 100 μl of EDTA-Plasma is incubated in a 1-step procedure with 150 μl labeled antibody on the microtiter plate for 1 h at 22 °C. After a 5-step washing process chemiluminescence is measured for 1 s per well by using the Centro LB 960 microtiter plate luminescence reader (Berthold Technologies).

The limit of detection of the assay was 3 pg/mL, while the limit of quantification, defining functional assay sensitivity, was 11 pg/ml. Thus this assay (11 to >1000 pg/ml) covers the entire spectrum of supranormal bio-ADM concentrations that have been reported to occur in pathological conditions [16]. Although this assay was originally developed using human samples the manufacturers determined 100% cross-reactivity of the assay with bio-ADM from animals, including pigs due to the evolutionarily highly conserved amino acid sequence of bio-ADM [16].

### Statistical analysis

Values are expressed as median and interquartile range (IQR). Group comparisons of continuous variables for individual time points were performed using the Kruskal–Wallis test. When Kruskal–Wallis test indicated a statistically significant difference and more than two groups were compared, post hoc pairwise comparisons were conducted using Dunn’s test. Changes over time within the same group were analyzed using the Wilcoxon signed-rank test. For both between-group and within-group multiple comparisons, *p*-values were adjusted using the Holm method. Since the primary objective was to assess the dynamics of bio-ADM plasma levels in hemorrhagic shock and their correlation with different treatment regimens—as well as with physiologic markers of macro- and microcirculation during treatment—we focused on evaluating changes in bio-ADM levels from T5 (after 60 min of untreated hemorrhagic shock) to subsequent treatment time points (T7: 60 min of treatment; T9: 120 min of treatment). To further explore associations between bio-ADM and individual hemodynamic or microcirculatory parameters, animals were additionally stratified into "low" and "high" subgroups based on a median split of the respective parameter at T9, and the resulting change in bio-ADM from T5 to T7 and T9 was compared between subgroups using the Wilcoxon rank-sum test. To examine whether bio-ADM during resuscitation was associated with subsequent macrohemodynamic status, we performed an exploratory multiple linear regression in the control and noradrenaline groups (n = 14). The fluid group was excluded from this model because, in the full cohort, treatment group and fluid volume are highly collinear — the fluid group was defined precisely by receiving substantially larger fluid volumes — which would preclude reliable separation of their respective effects on MAP. MAP at T9 was modeled as the dependent variable, with log2-transformed bio-ADM at T7 as the main predictor and cumulative fluid volume and treatment group as covariates. Two sensitivity analyses (examining the contemporaneous MAP–bio-ADM association at T9, and the effect of including the fluid group) are reported in the Supplementary Material. Regression coefficients are reported with 95% CIs and p-values, and model fit was assessed using R^2^ and adjusted R^2^. All statistical tests were 2-tailed and a two-sided p-value of <0.05 was considered for significance. The statistical analyses were performed using R version 4.2.2 (http://www.r-project.org, library rms, Hmisc, ROCR) and the Statistical Package for the Social Sciences (SPSS) version 22.0 (SPSS Inc., Chicago, Illinois, USA).

## Results

This secondary analysis included 20 animals (weight 35.5 [34–37.5] kg). Of the 27 animals in the original study, three died, two were excluded due to altered baseline hemodynamic parameters, and blood samples were unavailable for two further animals. The control group (CG) consisted of 7 animals, the noradrenaline group (NG) of 7 animals, and 6 animals were included in the fluid group (FG).

### Macro- and microcirculatory effects of resuscitation strategies

In this cohort, representing a subgroup with available bio-ADM measurements within a previously described hemorrhagic shock model [13], baseline hemodynamics were comparable between groups and remained stable during surgical preparation. Hemorrhage consistently induced a pronounced shock state with severe hypotension, tachycardia, and rising lactate levels.

Cumulative fluid administration increased from T5 to T9 in all groups (overall: 1779 [1638.75- 1992.5] ml to 3140 [2590.50–5862.5] ml; see additional file 1A). At T5, cumulative volumes were comparable (FG: 1890 [1713.75–2040] ml; NG: 1650 [1531–1773.5] ml; CG: 1940 [1779–2245] ml; p=0.16). By T9, administered cumulative fluid volumes differed significantly between groups (p=0.001), driven by substantially higher administration in FG (7042.5 [6086.25–7612.5] ml) compared to NG (2505 [2451–2716] ml) and CG (2840 [2689–3275] ml) (FG vs. NG: p<0.001; FG vs. CG: p=0.02; CG vs. NG: p=0.22).

Noradrenaline was administered exclusively in NG and increased from T7 to T9 (1.41 [1.33–1.52] to 2.08 [1.91–2.19] µg/kg/min, see additional file 1B).

**Fig. 2:**
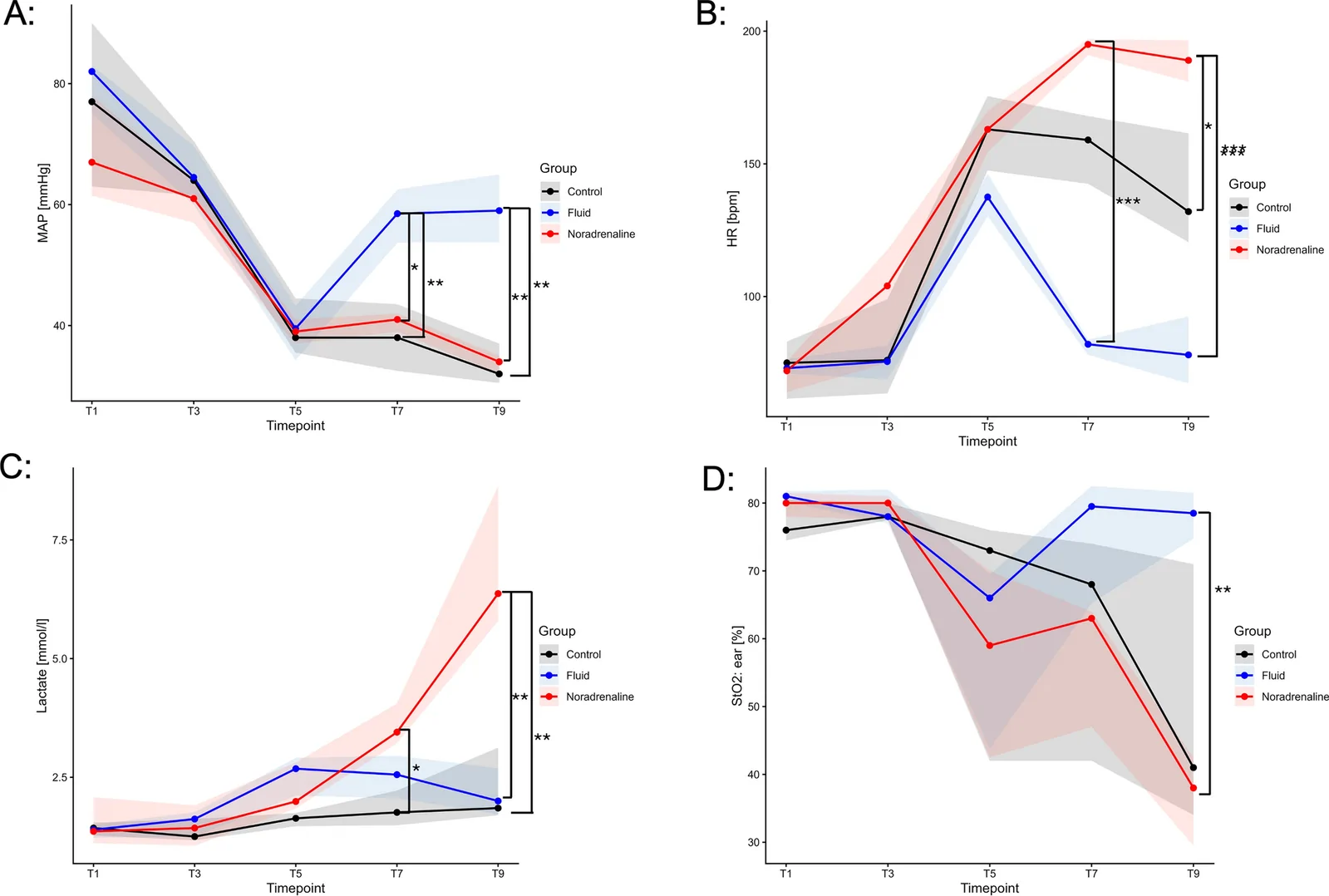
Time course of macrohemodynamic parameters (**A**: MAP, **B**: Heart Rate) and microcirculatory surrogates (**C**: lactate, **D**: tissue oxygenation at ear) during the experiment. Data are presented as median (points) with interquartile range (shaded areas), shown separately for experimental groups as indicated in the legend. T5 denotes 60 min of blood withdrawal, immediately prior to intervention. T7 and T9 represent 60 and 120 min after intervention onset, respectively. Statistical significance between groups was assessed using the Kruskal–Wallis test followed by Dunn’s post hoc test with Holm correction for multiple comparisons;* p<0.05, ** p<0.01, *** p<0.001

At T9, treatment groups showed clearly divergent resuscitation patterns (Fig. 2): FG achieved partial hemodynamic stabilization, whereas NG remained in a profound shock state. This was reflected by higher MAP in FG (overall p=0.002; FG vs. NG p=0.006; FG vs. CG p=0.005, NG vs CG p=0.8) and lower heart rate (overall p<0.001; FG vs. NG p<0.001; FG vs. CG p=0.09; NG vs. CG p=0.038), although the predefined MAP target (> 65 mmHg) was not consistently achieved in any group.

These macrohemodynamic differences were paralleled by alterations in tissue oxygenation markers (Fig. 2C, D). At T9, lactate levels were significantly higher in NG compared to FG and CG (overall p=0.002; FG vs. NG p=0.007; FG vs. CG p=0.95; NG vs. CG p=0.005). Furthermore, lactate increased significantly over time exclusively in NG from T5 to T9 (p=0.045), indicating ongoing tissue hypoperfusion. Consistently, direct assessment of the microcirculation via StO₂ Ear increased in FG but decreased in NG (overall p=0.006; FG vs. NG p=0.005; CG vs. FG p=0.07; CG vs. NG p=0.27), supporting preserved microcirculatory oxygenation under fluid resuscitation and impaired oxygen delivery under vasopressor therapy.

Both central venous oxygen saturation (ScvO₂) and oxygen delivery (DO₂) declined markedly following shock induction (T5) in all groups. During resuscitation, ScvO₂ recovered in the fluid group but continued to decline in the control and noradrenaline groups (overall p<0.001), whereas DO₂ declined similarly across all groups without significant between-group differences thereafter. Full time courses for these, as well as further microcirculatory HSI parameters, are provided in the supplementary material (see additional files 2, 3 and 4).

### bio-ADM dynamics across treatment groups

Overall bio-ADM concentrations measured in this study ranged from 2.2 pg/ml to 98.2 pg/ml.

**Fig. 3:**
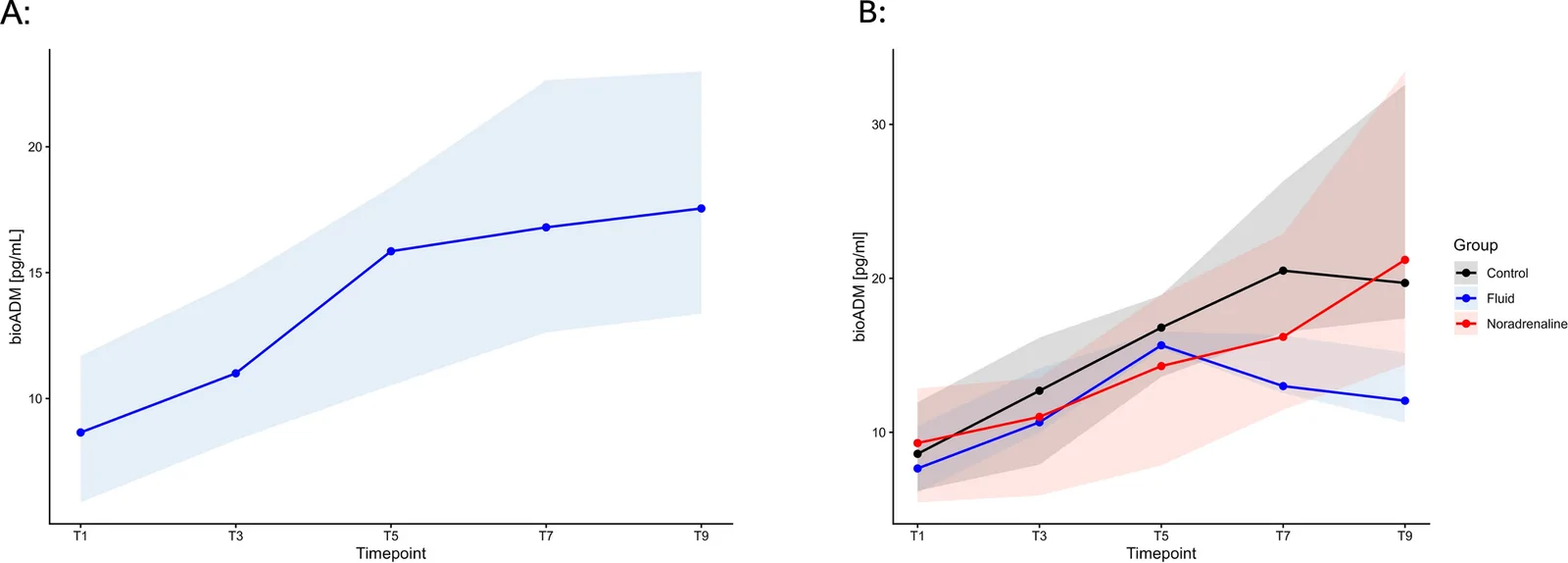
Time course of bio-ADM plasma levels during the entire experiment. (**A**) Bio-ADM plasma levels of all animals analyzed together (*n* = 20). (**B**) Bio-ADM plasma levels stratified by group: control group (*n* = 7) and intervention groups (fluid, n = 6; noradrenaline, n = 7), shown separately as indicated in the legend. Data are presented as median (points) with interquartile range (shaded areas), in B shown separately for experimental groups as indicated in the legend

A statistically significant rise of bio-ADM levels from T1 to T9 could be seen once all pigs were analyzed together (Fig. 3, p<0.001). Bio-ADM levels increased from T1 to T3 during anesthesia induction and abdominal surgery. Following shock induction at T3, a further and sustained increase in bio-ADM concentrations was observed over time. Highest bio-ADM levels were measured at T9 (17.55 [13.38–23.00] pg/ml).

Separation into different treatment groups showed a continued rise in bio-ADM levels in the NG throughout the treatment phase, whereas bio-ADM levels in the CG rose during the shock and early treatment phase and then plateaued; in the fluid group, bio-ADM increased after shock induction and remained stable during the treatment phase. Bio-ADM plasma levels were highest at T9 in CG (19.70±15.20pg/ml) and NG (21.20±19.05pg/ml). In the FG, bio-ADM plasma levels rose after shock induction at T3 to 15.65±1.13pg/ml at T5 and remained stable through T7 (13.00±1.13pg/ml) and T9 (12.05±4.52pg/ml). At T9, the fluid group´s bio-ADM change from T5 was significantly lower than in the noradrenaline group (p=0.003) and the control group (p=0.022).

### Association of bio-ADM plasma levels and markers of macrocirculation

**Fig. 4:**
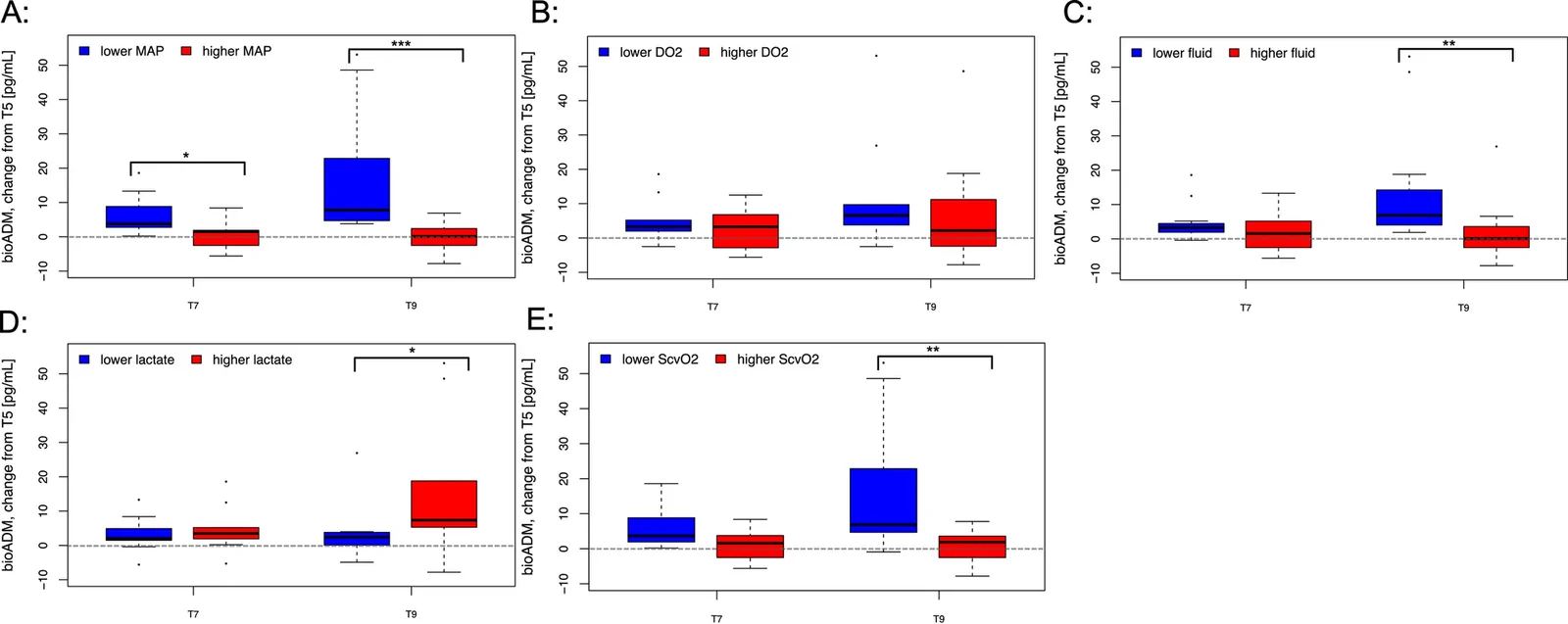
Bio-ADM changes stratified by T9-based median split of hemodynamic, metabolic, and resuscitation parameters. Change in bio-ADM plasma levels from T5 to T7 and from T5 to T9 in all animals. Animals were stratified based on a median split of the respective parameter measured at T9, and this fixed stratification was applied to both time intervals to visualize temporal changes: (**A**) mean arterial pressure (MAP), (**B**) oxygen delivery (DO₂), (**C**) fluid administration, (**D**) lactate, and (**E**) central venous oxygen saturation (ScvO₂). Values are presented as median with interquartile range (IQR), measured in pg/mL. *p<0.05, **p<0.01

Overall, bio-ADM levels increased most prominently in animals with lower MAP, lower fluid administration, higher lactate, or lower ScvO₂ (Fig. 4).

Dividing animals by median MAP at T9 revealed a significantly smaller increase in bio-ADM levels from T5 to T9 in pigs with higher MAP (p<0.001). No relationship was observed between DO₂ and bio-ADM levels. Animals receiving higher fluid volumes (> 3000 mL) at T9 showed a significantly smaller increase in bio-ADM levels from T5 to T9 compared to those with lower fluid administration (p=0.01) (Fig. 4).

Pigs with higher lactate levels at T9 exhibited a significant increase in bio-ADM from T5 to T9 compared to animals with lactate below 2.75mmol/L (p=0.02), whereas no difference was observed at T7 (p=0.5). Similarly, stratification by central venous oxygen saturation (ScvO₂) at T9 revealed a significant association: animals with ScvO₂ below 51% demonstrated a pronounced increase in bio-ADM compared to those with ScvO₂ above 51% (p=0.004) (Fig. 4).

To evaluate to what extend the associations observed for bio-ADM were confounded by differences in fluid resuscitation, we repeated the median-split analysis after excluding animals from the FG, thereby comparing only the CG and the NG (n=14). For MAP, the inverse association with bio-ADM change was preserved and remained statistically significant at T7, though it no longer reached significance at T9. A similar inverse trend was observed for ScvO₂, with lower ScvO₂ associated with higher bio-ADM increases at both time points, although this did not reach statistical significance. No significant associations were observed between bio-ADM and DO₂ or arterial lactate in this restricted cohort. Fluid administration itself showed no apparent association with bio-ADM levels in this restricted analysis (see additional file 5). To further test for an independent association between bio-ADM and hemodynamic status, we performed a multiple linear regression analysis in the same subgroup. Complete data were available for 14 animals. The overall model explained 68.8% of the variance in MAP at T9 (adjusted R^2^ = 0.594) and was statistically significant (F[3,10] = 7.35, p = 0.0069). After adjustment for cumulative fluid administration and treatment group, each doubling of bio-ADM at T7 was associated with a 4.62 mmHg lower MAP at T9 (β = −4.62 mmHg, 95% CI −6.90 to −2.35; p = 0.0011; Table 1). Adding bio-ADM to the model increased R^2^ from 0.048 to 0.688 (ΔR^2^ = 0.640; partial F = 20.50; p = 0.0011). Neither cumulative fluid volume (p = 0.163) nor assignment to the noradrenaline group (p = 0.976) was associated with MAP at T9. Results of the sensitivity analyses are presented in the Supplementary Material (Table S1).

**Table 1:** Primary multiple linear regression analysis of MAP at T9 in the control and noradrenaline groups

**Predictor (scale or contrast)**	**β, mmHg**	**95% CI**	**p value**
Log2 bio-ADM at T7 (per doubling)	-4.62	-6.90 to − 2.35	0.001
Cumulative fluid volume to T9 (per 1,000 mL)	2.55	-1.23 to 6.33	0.163
Noradrenaline group (vs control)	0.06	-4.31 to 4.43	0.976

Unstandardized ordinary least-squares coefficients are shown; the intercept is omitted. The outcome was MAP at T9. *n* = 14; F(3,10) = 7.35, model p = 0.0069; R^2^ = 0.688; adjusted R^2^ = 0.594; RMSE = 2.80 mmHg. Adding log2 bio-ADM increased R^2^ by 0.640 (partial F = 20.50, p = 0.0011). Control was the reference group. CI, confidence interval; MAP, mean arterial pressure; RMSE, root mean squared error

### Improved tissue oxygenation assessed by HSI is associated with lower bio-ADM levels

Bio-ADM levels increased predominantly in animals with impaired StO₂ and microcirculatory perfusion, whereas higher TWI was associated with a smaller rise.

**Fig. 5:**
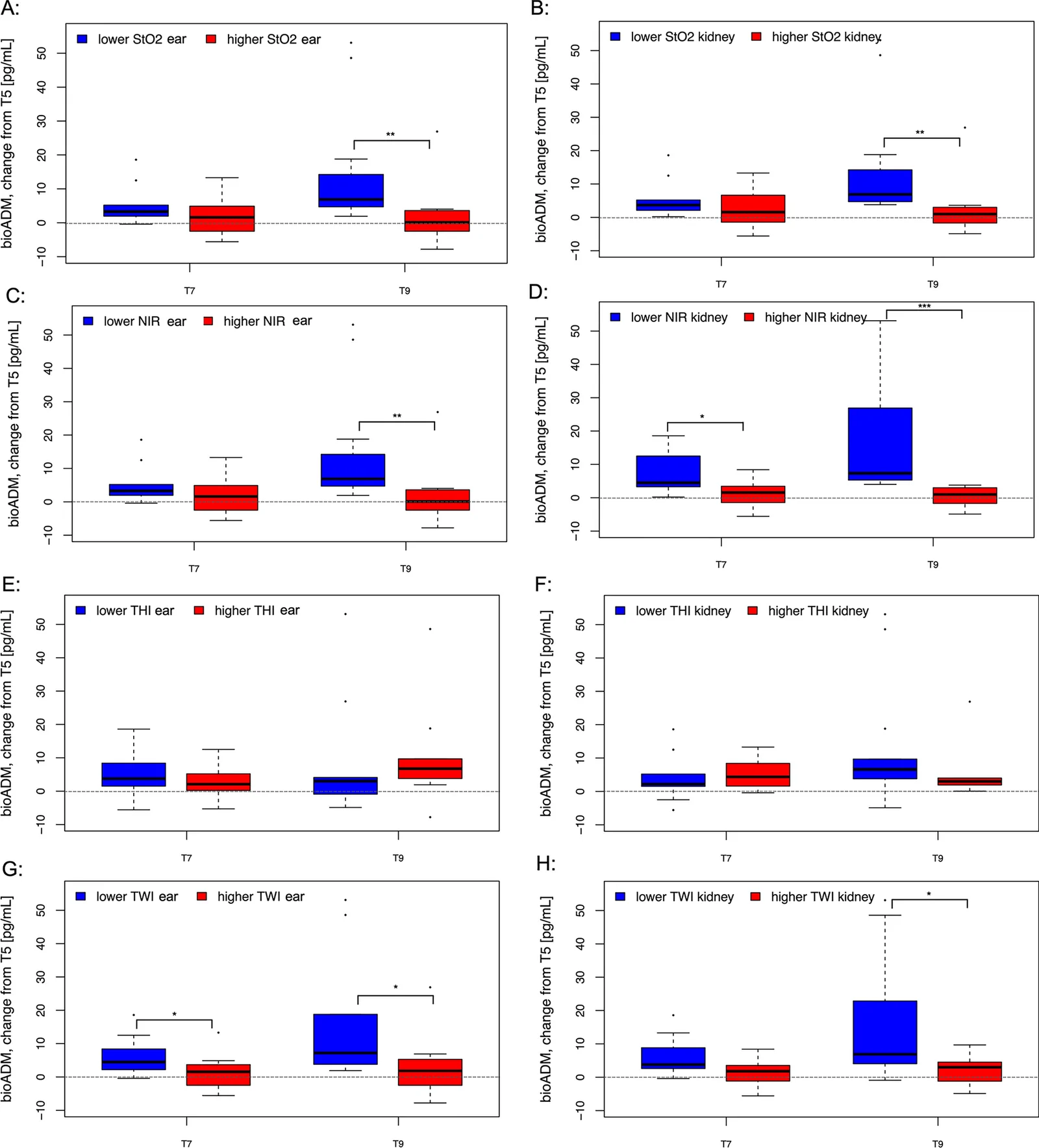
Bio-ADM changes stratified by T9-based median split of tissue perfusion parameters. Change in bio-ADM plasma levels from T5 to T7 and from T5 to T9 in all animals. Animals were stratified based on a median split of the respective parameter measured at T9, and this fixed stratification was applied to both time intervals to visualize temporal changes. (**A**) Tissue oxygenation (StO₂) of ear and (**B**) of kidney, (**C**) near-infrared perfusion index (NIR) of ear and (**D**) of kidney, (**E**) tissue hemoglobin index (THI) of ear and (**F**) of kidney, (**G**) tissue water index (TWI) of ear and (**H**) of kidney. Bio-ADM levels are presented as median with interquartile range (IQR), measured in pg/ml. StO₂ is expressed as percentage; NIR, THI, and TWI are dimensionless index values (arbitrary units). * p<0.05, ** p<0.01, *** p<0.001

Stratification of animals based on StO₂ at ear and kidney at T9 revealed a moderate decrease in bio-ADM levels from T5 to T9 in subgroups with higher StO₂ values. Animals with StO₂ Ear ≥58.5% showed a tendency for decreasing bio-ADM levels over time, whereas those with StO₂ Ear <58.5% exhibited a marked increase from T5 to T9. Similarly, animals with StO₂ Kidney <56% displayed a pronounced rise in bio-ADM plasma concentrations over the same period. Median-split analysis at T9 confirmed a statistically significant effect on the trajectory of bio-ADM levels, with p=0.006 for StO₂ Ear and p=0.004 for StO₂ Kidney (Fig. 5 A, B). Consistent with these findings, peripheral microcirculatory values assessed by NIR showed similar associations. At T9, animals with lower ear NIR values (≤ 39.5%) demonstrated a significantly greater increase in bio-ADM levels from T5 compared to animals with higher NIR (> 39.5%, p=0.006). Likewise, animals with lower kidney NIR values (≤ 16%) exhibited a significantly larger rise in bio-ADM compared to those with higher NIR (> 16%), with differences reaching significance at both T7 (p = 0.03) and T9 (p<0.001) (Fig. 5 C, D).

No significant differences were observed in bio-ADM trajectories from T5 to T9 when animals were grouped according to THI at ear or kidney. Notably, THI values were consistently higher in kidney than at ear (Fig. 5 E, F).

In contrast, higher TWI at ear and kidney at T9 was associated with a significantly smaller increase in bio-ADM levels from T5 to T7 and T9 compared to animals with lower TWI. This effect reached statistical significance at T9 for both TWI Ear (p=0.04) and TWI Kidney (p=0.03), and TWI Ear was already significantly associated with bio-ADM changes at T7 (p=0.03) (Fig. 5 G, H).

When restricting this analysis to animals of the CG and NG only, a similar trend was observed for measures of tissue oxygenation, with animals showing impaired StO₂ and NIR values at T9 tending to show higher bio-ADM levels, consistent with the pattern seen in the full cohort, although this reached statistical significance only for NIR at the kidney at T9. No discernible trend in bio-ADM levels was observed when animals were stratified by THI or TWI at either ear or kidney (see additional file 6).

## Discussion

In this porcine model, we observed a significant increase in bio-ADM concentrations during hemorrhagic shock. Adequate resuscitation with fluid therapy was associated with a stabilization, rather than a clear decrease, of circulating bio-ADM levels. As discussed below, this pattern is consistent with—but does not prove—a biological effect of resuscitation on bio-ADM kinetics, given the potential contribution of hemodilution in the fluid group.

In clinical studies, bio-ADM concentrations increase with disease severity, ranging from <30pg/mL in healthy individuals to markedly higher levels in cardiogenic and septic shock [10, 11, 17–19].

A modest bio-ADM increase occurred between T1 and T3 during anesthesia and surgery. Endothelial injury is a major trigger for bio-ADM release and explains the high levels seen in septic shock, where bio-ADM acts protectively to maintain endothelial barrier function [7, 20]. Surgical manipulation and inflammation mediated by IL-6 contribute to endothelial disruption. Gregersen et al. [21] and Steppan et al. [22] demonstrated that IL-6 correlates with markers such as syndecan-1 and sVEGFR-1. The modest perioperative bio-ADM increase in our study aligns with findings of Otao et al. [23].

Following hemorrhage induction (T5), bio-ADM concentrations increased further and remained elevated throughout the resuscitation period. Severe blood loss causes systemic endothelial injury—termed “endotheliopathy of trauma”- driven by inflammation, sympathetic activation, and ischemia, all contributing to glycocalyx degradation [2, 24, 25]. The concept of “shock-induced endotheliopathy” (SHINE) describes a similar process independent of shock etiology [26]. Given the endothelial-stabilizing properties of bio-ADM, the sustained increase observed in our model is consistent with a compensatory response to ongoing endothelial stress during hemorrhagic shock.

Bio-ADM trajectories differed between study groups from T5 onward. Levels continued to rise in the noradrenaline group throughout the treatment phase, rose in the control group from T5 to T7 before plateauing thereafter, and stabilized in the fluid group already from T5 onward. As shown previously, only the fluid group achieved hemodynamic stabilization with restored cardiac output, improved mean arterial pressure, and enhanced StO₂ measured by hyperspectral imaging. In contrast, neither control nor noradrenaline groups reached MAP targets, and noradrenaline further impaired oxygenation [13]. As hemorrhagic shock is a form of hypovolemic shock, effective volume resuscitation directly addresses the underlying pathophysiology, and the attenuated rise of bio-ADM in the fluid group occurred in temporal association with hemodynamic recovery. However, this group also received markedly larger cumulative fluid volumes than the control and noradrenaline groups (approximately three-fold higher by T9), and hemodilution therefore represents a major potential confounder that cannot be separated from a genuine biological stabilization of endothelial injury using our data. Both mechanisms likely contributed to the observed pattern, and their relative contributions cannot be quantified in this dataset.

Animals with higher MAP at T9 showed a significantly smaller increase in bio-ADM from T5 to T9 than those with lower MAP. As persistent hypotension can compromise tissue perfusion pressure and thereby contribute to endothelial stress, this pattern is consistent with ongoing endothelial dysfunction described by SHINE. In the noradrenaline group, persistently elevated bio-ADM may also reflect catecholamine-induced endothelial stress, as catecholamines promote glycocalyx shedding [25, 27]. Paradoxically, bio-ADM itself induces vasodilation via cAMP- and NO-dependent pathways, potentially lowering MAP further and perpetuating shock [28, 29]. Thus, bio-ADM may act both as a marker and mediator of hemodynamic deterioration. As MAP was closely tied to treatment allocation in this model—animals with higher MAP at T9 were disproportionately drawn from the fluid group—this relationship could partly reflect treatment assignment rather than a direct hemodynamic effect. To test this, we restricted the analysis to the control and noradrenaline groups only (n=14): The inverse MAP–bio-ADM association remained statistically significant at T7 and persisted as a directional trend at T9, though no longer reaching significance at this later time point – supporting that the relationship is not solely an artifact of grouping by treatment. This was further corroborated by a multiple linear regression analysis in the same subgroup: each doubling of bio-ADM at T7 was associated with an approximately 4.6 mmHg lower MAP at T9, independent of cumulative fluid administration and treatment group; fluid volume itself was not associated with MAP in this population, suggesting that variation in administered volume alone did not account for the observed bio-ADM–MAP relationship. A concurrent, reverse-direction analysis within the same subgroup similarly showed an inverse association between MAP and bio-ADM at T9 after adjustment for fluid administration, supporting the consistency of this relationship within the control and noradrenaline groups. When the fluid group was included, this association was attenuated and no longer significant, while fluid volume became strongly associated with MAP instead. As administered fluid volume only imperfectly captures actual plasma dilution, this model cannot reliably separate dilution-related effects from effects due to sufficient resuscitation, and the primary association therefore cannot be generalized to animals receiving high-volume fluid resuscitation. Overall, these findings support elevated bio-ADM as a marker of persistent hemodynamic and endothelial stress, though the small sample size and diverging results after including the fluid group warrant cautious interpretation.

Previous clinical studies have reported similar associations. Tolppanen et al. [10] found an inverse MAP–bio-ADM relationship in cardiogenic shock, while Mebazaa (2018) linked high bio-ADM to greater vasopressor demand in sepsis. Takagi et al. [30] did not confirm this in cardiogenic shock, emphasizing multifactorial bio-ADM regulation in critical illness.

However, normalization of MAP does not ensure adequate perfusion. Microcirculatory dysfunction may persist despite restored systemic parameters [31]. In our study, elevated bio-ADM was associated with high lactate and low central venous oxygen saturation (ScvO₂), but not with oxygen delivery (DO₂)—a dissociation observed in both the full cohort and the CG/NG-only sensitivity analysis. This argues against a simple, direct link between global oxygen supply–demand mismatch and bio-ADM release: while low ScvO₂, an integrative parameter also shaped by microcirculatory shunting and metabolic demand, was associated with higher bio-ADM, DO₂ alone was not. The ScvO₂–bio-ADM association was strongest in late shock phases, consistent with reports linking high bio-ADM to therapy-refractory cardiogenic shock and organ failure [10, 30]. We therefore interpret elevated bio-ADM primarily as a marker of endothelial and microcirculatory stress rather than as a direct readout of macrocirculatory oxygen transport.

HSI provided real-time insights into the microcirculation. Reduced ear and renal oxygenation correlated with higher bio-ADM concentrations, indicating a potential diagnostic role for bio-ADM in early tissue hypoperfusion. With its short half-life (~ 22 min), bio-ADM may rapidly reflect resuscitation efficacy [32]. The strong association between renal oxygenation and bio-ADM is particularly relevant, as renal hypoperfusion is a major driver of shock-induced acute kidney injury a major complication of hemorrhagic shock and predictor of ICU morbidity and mortality [33]. Bio-ADM may therefore serve as an early biomarker of renal compromise. Similarly, when restricting this analysis to the control and noradrenaline groups only, impaired tissue oxygenation (StO₂ and NIR) at T9 remained associated with a trend towards higher bio-ADM levels, consistent with the pattern observed in the full cohort, this association reached statistical significance for NIR at the kidney, while the remaining comparisons did not reach significance in this reduced subgroup. Combined with the corresponding trend observed for MAP (see above), these findings provide supportive evidence that the associations between hemodynamic/microcirculatory impairment and bio-ADM release are unlikely to be fully explained by dilution in the fluid group alone (see Limitations for a more detailed discussion of the potential dilutional contribution).

The TWI closely reflected volume administration [13]. Consistent with this significant increase in TWI, we observed a mitigated rise in bio-ADM concentrations in the fluid group throughout the treatment phase (T5–T9), which may reflect reduced endothelial injury through effective resuscitation. However, excessive fluid after intravascular restoration can promote edema, increase diffusion distances, and impair oxygenation. Notably, in the later phase (T7–T9), bio-ADM reached a plateau while TWI continued to rise—most likely reflecting ongoing fluid administration itself, as TWI directly tracks tissue water content. This dissociation does not allow us to infer a causal relationship between bio-ADM and TWI in either direction,our data cannot determine whether the two are mechanistically linked or simply co-occur as parallel consequences of the resuscitation strategy.

The importance of preserving endothelial integrity in critical illness is increasingly recognized. In trauma, glycocalyx degradation has been linked to increased mortality (Johansson et al. 2011). Severe trauma–associated cardiac failure (STAC) has recently been identified as an additional contributor to persistent hypotension, impaired perfusion, and increased mortality in severe trauma [34]. Multifactorial reductions in cardiac output, largely driven by inflammation, can trigger bio-ADM release, as shown in acute myocardial infarction and cardiogenic shock, where bio-ADM reliably predicts mortality [10, 35]. Elevated bio-ADM may thus reflect the combined burden of endothelial injury, hypoperfusion, and cardiac dysfunction, serving as a dynamic biomarker to guide individualized resuscitation when combined with microcirculatory monitoring. However, its lack of specificity for any single mechanism or shock etiology is likely to limit its use as a standalone diagnostic or monitoring tool, and whether bio-ADM adds clinically actionable information beyond established markers of perfusion and endothelial injury remains unclear. Whether, and by which specific therapeutic actions, bio-ADM could meaningfully guide individualized resuscitation was not addressed by the present data and warrants dedicated investigation. Extrapolating from associations reported in septic shock, bio-ADM may also carry prognostic value in hemorrhagic shock [36, 37],the inverse association between bio-ADM at T7 and MAP at T9 observed in our multiple regression analysis in the control and noradrenaline groups may offer a preliminary hint in this direction, although this exploratory finding requires confirmation in larger, prospective cohorts before any prognostic value can be inferred.

This study has several limitations. As a porcine model, translational applicability to humans is limited. The analysis was based on secondary data from an experiment not designed to assess bio-ADM kinetics, with restricted sampling intervals and a small overall sample size (*n* = 20). No group undergoing anesthesia and surgery without hemorrhagic shock was included, preventing separation of anesthesia and surgery effects from shock. Direct markers of endothelial or glycocalyx injury (e.g., syndecan-1, sVEGFR-1) were not measured. Organ dysfunction could only be inferred indirectly, as renal functional endpoints were unavailable, and mortality was not assessed due to the timeline of the experiments, precluding evaluation of potential correlations between bio-ADM and prognosis. As discussed above, a dilutional contribution to the observed bio-ADM stabilization in the fluid group cannot be fully excluded. Such hemodilution could in principle be estimated from changes in hematocrit, but a reliable correction was not feasible here, given the small size of the fluid group (n = 6) and incomplete hematocrit sampling across time points. Multiple linear regression models showed significant association between bio-ADM and future macrohemodynamic parameters. However, the direction of the association remains the same including fluid resuscitation group but failed to reach statistical significance.

Despite these limitations, our findings suggest that bio-ADM reflects endothelial and microcirculatory stress in hemorrhagic shock and may track the trajectory of shock and resuscitation, although its lack of specificity and the unresolved contribution of hemodilution limit the strength of this evidence. Whether bio-ADM offers therapeutic utility beyond established markers of perfusion and endothelial injury remains to be established. Future prospective studies, in both animal models and humans — ideally with designs able to separate dilutional from biological effects — are needed to validate bio-ADM as a diagnostic biomarker and clarify its potential role in guiding individualized resuscitation.

## Conclusions

In summary, our findings are consistent with the hypothesis that bio-ADM rises during hemorrhagic shock and reflects an ongoing state of hemodynamic and microcirculatory compromise, rather than indicating a specific underlying disease process. Effective resuscitation appeared to be temporally associated with a stabilization of bio-ADM levels, suggesting that bio-ADM may track the trajectory of shock and its resolution, although a contribution of hemodilution to this pattern cannot be fully excluded. Overall, our data support bio-ADM as a comparatively unspecific marker of endothelial stress associated with hypoperfusion, potentially applicable across various shock states rather than being specific to a single etiology such as sepsis or cardiogenic shock. Regular measurement of bio-ADM may thus provide complementary, real-time information on the shock induced endotheliopathy, adequacy of resuscitation and tissue perfusion, though further prospective studies are needed to confirm these observations.

## Supplementary Information

Supplementary Material 1.

Supplementary Material 2.

Supplementary Material 3.

Supplementary Material 4.

Supplementary Material 5.

Supplementary Material 6.

Supplementary Material 7.

## Data Availability

The datasets used and/or analysed during the current study are available from the corresponding author on reasonable request.

## Abbreviations

ADM: Adrenomedullin
Bio-ADM: Bio-Adrenomedullin
cAMP: Cyclic adenosine monophosphate
DO_2_: Oxygen delivery
HSI: Hyperspectral imaging
IL-6: Interleukin 6
ICU: Intensive care unit
IQR: Interquartile range
MAP: Mean arterial pressure
NO: Nitric oxide
PiCCO: Pulse contour cardiac output
StO₂: Tissue oxygenation
NIR: Near infrared
THI: Tissue hemoglobin index
TWI: Tissue water index
ScvO_2_: Central venous oxygen saturation
SHINE: Shock-induced endotheliopathy
sVEGFR1: Soluble vascular endothelial growth factor receptor 1
